# Recommendations for preventing the spread of vancomycin resistance. Hospital Infection Control Practices Advisory Committee.

**DOI:** 10.3201/eid0102.952010

**Published:** 1995


### WHONET: An Information System for Monitoring Antimicrobial Resistance

WHONET is an information system developed to support The World Health Organization's (WHO) goal of global surveillance of bacterial resistance to antimicrobial agents. Microbiologists, clinicians and infection control workers may use its software to enhance monitoring of drug resistance in their hospitals and communities and to merge their files into national, regional, and global networks for surveillance of drug resistance. WHONET software can be installed on personal computers and be configured for the locations of the patients a laboratory serves and for the antimicrobial agents it tests. The program accepts susceptibility test results and allows printing of reports and logbooks and retrieval of data. If the laboratory already has a computerized reporting system, a translation program can be created to download the laboratory's files into WHONET. Either way, the microbiologists and other infectious disease specialists gain new analytical tools to monitor and manage susceptibility test quality and the spread of drug resistance locally and outside their area.

WHONET can also analyze stored data. From a single screen, a WHONET user selects the type of analysis to run, the species of bacteria to analyze, the subsets of isolates to include (e.g., all, isolates from urine only, and isolates resistant to gentamicin and from certain locations), and the antimicrobial agents and period to examine. Types of analyses include percentage of data categorized as resistant, intermediate, or susceptible by standard or other breakpoints; distributions of test measurements (zone diameter, minimal inhibitory concentration) in the form of histograms; scatterplots comparing measurements for different agents or methods for the same isolates; and line listings of isolates grouped by combinations of agents to which they are resistant (antibiotypes) to trace distinctive strains. Isolates with uncommon antibiotypes can also be flagged on entry so that they may be rechecked while still available, and local outbreaks can be detected early.

Although test results are entered and monitored locally on software configured for local use, they are filed in a universal file format so that any copy of the program can analyze the files of any laboratory. This feature has enabled groups of users in 10 countries to set up passive surveillance systems by pooling and analyzing their files collaboratively. WHONET assists such initiatives by providing file encryption options to ensure confidentiality before data are pooled and analyzed.

Ongoing local analysis by local workers is the foundation of the system. It detects local problems in testing, which no laboratory can avoid entirely, and thus improves the overall quality of the files. It delineates local spread of drug-resistant strains, which aids infection control and can explain and correct uncommon prevalence of certain types of drug resistance at certain sites. It allows local workers to distinguish their problems from those of other sites and focus on infection control or antimicrobial use that might be related to those problems.

Expansion of the system has been recommended by the WHO Scientific Working Group on Monitoring and Management of Bacterial Resistance to Antimicrobial Agents. For more information or for participation contact

**Thomas F. O'Brien and John M. Stelling**
WHO Collaborating Center for Surveillance of
Resistance to Antimicrobial Agents
Microbiology, Brigham and Women's Hospital
Boston, MA 02115, USA
tel (1-617) 732-6803
fax (1-617) 732-4144
Internet: whonet@bustoff.bwh.harvard.edu

### Recommendations for Preventing the Spread of Vancomycin Resistance

CDC's Hospital Infection Control Practices Advisory Committee (HICPAC) has published "Recommendations for Preventing the Spread of Vancomycin Resistance." The recommendations focus on vancomycin-resistant enterococci (VRE).

The reported incidence of infection and colonization with VRE in U.S. hospitals has increased rapidly in the last 5 years. This increase has compounded the need for antimicrobial drugs to treat VRE infections. Most VRE are also resistant to multiple other drugs (e.g., aminoglycoside and ampicillin), which have been used for treating VRE infections. In addition, the possibility that the vancomycin-resistance genes present in VRE may be transferred to other gram-positive microorganisms, especially *Staphylococcus aureus*, is a serious public health concern.

Although the epidemiology of VRE has not been fully elucidated, and most enterococcal infections have been attributed to the patient's endogenous flora, recent studies have demonstrated that enterococci, including VRE, can be spread directly from patient to patient or indirectly by transient carriage on the hands of personnel or contaminated environmental surfaces and patient-care equipment.

In its recommendations, HICPAC stresses that the prevention and control of vancomycin resistance will require a coordinated, concerted effort from various departments of a hospital. Because the rec-

